# Solitary Fibrous Tumor of Neck Mimicking Cold Thyroid Nodule in 99m Tc Thyroid Scintigraphy

**DOI:** 10.1155/2013/805745

**Published:** 2013-10-01

**Authors:** Oya Topaloglu, Bekir Ucan, Taner Demirci, Muyesser Sayki Arslan, Guleser Saylam, Evrim Onder, Sinan Gultekin, Alper Dilli, Mustafa Sahin, Erman Cakal, Mustafa Ozbek, Tuncay Delibasi

**Affiliations:** ^1^Department of Endocrinology and Metabolism, Diskapi Yildirim Beyazit Training and Research Hospital, 06130 Ankara, Turkey; ^2^Department of Otorhinolaryngology, Diskapi Yildirim Beyazit Training and Research Hospital, 06130 Ankara, Turkey; ^3^Department of Pathology, Diskapi Yildirim Beyazit Training and Research Hospital, 06130 Ankara, Turkey; ^4^Department of Nuclear Medicine, Diskapi Yildirim Beyazit Training and Research Hospital, 06130 Ankara, Turkey; ^5^Department of Radiology, Diskapi Yildirim Beyazit Training and Research Hospital, 06130 Ankara, Turkey; ^6^Department of Endocrinology and Metabolism, Ankara University School of Medicine, 06450 Ankara, Turkey

## Abstract

A 68-year-old man had a rapidly growing, painless neck mass, thought to be nodular goiter. Ultrasonography showed a giant, heterogeneous mass occupying the middle and superior poles and protruding outside of the left thyroid lobe. The results of the thyroid function tests were normal. Thyroid scintigraphy revealed a large hypoactive nodule in the left thyroid lobe. Complete surgical removal of tumor was performed and macroscopically demonstrated a well-demarked lesion outside the thyroid gland. Microscopically, the lesion was composed of fibroblast-like spindle cells in a patternless architecture and extensive stromal hyalinization. Immunohistochemistry showed positive reaction for CD34 in spindle cells and diffuse bcl-2 staining. The pathology was confirmed as solitary fibrous tumor. In the follow-up period after surgery, thyroid scintigraphy showed normal left thyroid lobe. Solitary fibrous tumor originated from or associated with thyroid gland is extremely rare. According to our knowledge, this is the first reported solitary fibrous tumor presenting like a cold thyroid nodule. This pathology must be considered for differential diagnosis of neck masses in the thyroid region.

## 1. Introduction

Solitary fibrous tumor (SFT) is a rare spindle cell neoplasm of mesenchymal origin that was first described in the pleura [1]. Since then, SFT has been recognized in various sites other than the pleura, such as mediastinum, pericardium, and peritoneum [2–4]. Head and neck SFTs are exceedingly rare, and they were firstly described as a case report in 1991 [5]. They were presented as asymptomatic slow-growing massess or with local symptoms due to compression. In most cases, complete surgical resection is the only appropriate treatment [6]. Diagnosis is often difficult and not definite until morphologic or immunohistochemical evaluation. SFT arising from the thyroid gland is also uncommon. It was first described by Taccagni et al. in 1993 [7]. Up to date and to the best of our knowledge, 23 cases of SFT in the thyroid gland have been reported in the literature, approximately all of them with benign characteristics [8–10], only 2 of them with malignant clinical features like local recurrence and metastasis [11]. Here, we present the first reported SFT mimicking hypoactive thyroid nodule in thyroid scintigraphy.

## 2. Case Report

**Figure 1:**
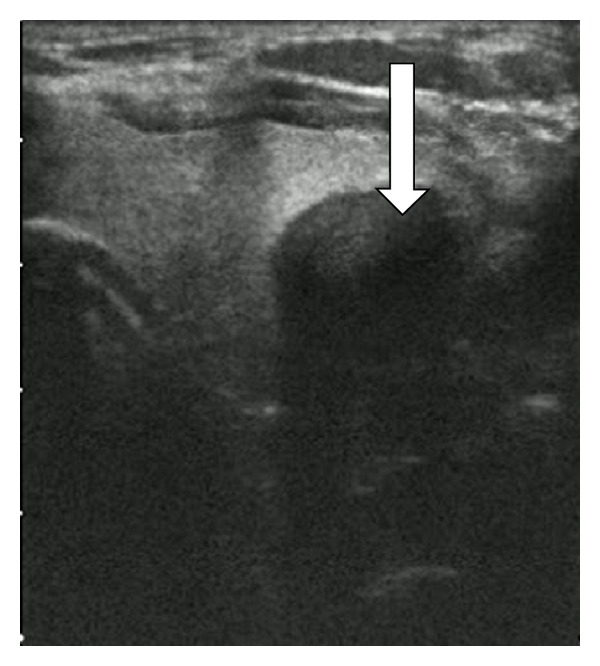
Ultrasonography showing a heterogeneously hypoechoic giant mass with undetermined inferior margins.

A 68-year-old man presented with a 2-month history of a rapidly growing mass in the left neck on the thyroid gland region. He did not have any history of thyroid disease. He did not describe any symptoms like hoarseness, dyspnea, local pain, or weight loss. In laboratory evaluation, thyroid function tests, serum calcium, phosphorus, and parathormone were all evaluated as normal ranges. Serum calcitonin was measured as <2 pg/mL, which is low. Thyroid ultrasound revealed a 5 mm isoechogenic nodule on the right lobe and an 8 cm in diameter hypoechogenic, heterogeneous giant mass with irregular margins that was thought to be a nonthyroidal mass compressing the thyroid left lobe or may be a mass originating from thyroid gland and protruding outside the gland (Figure 1). 

**Figure 2:**
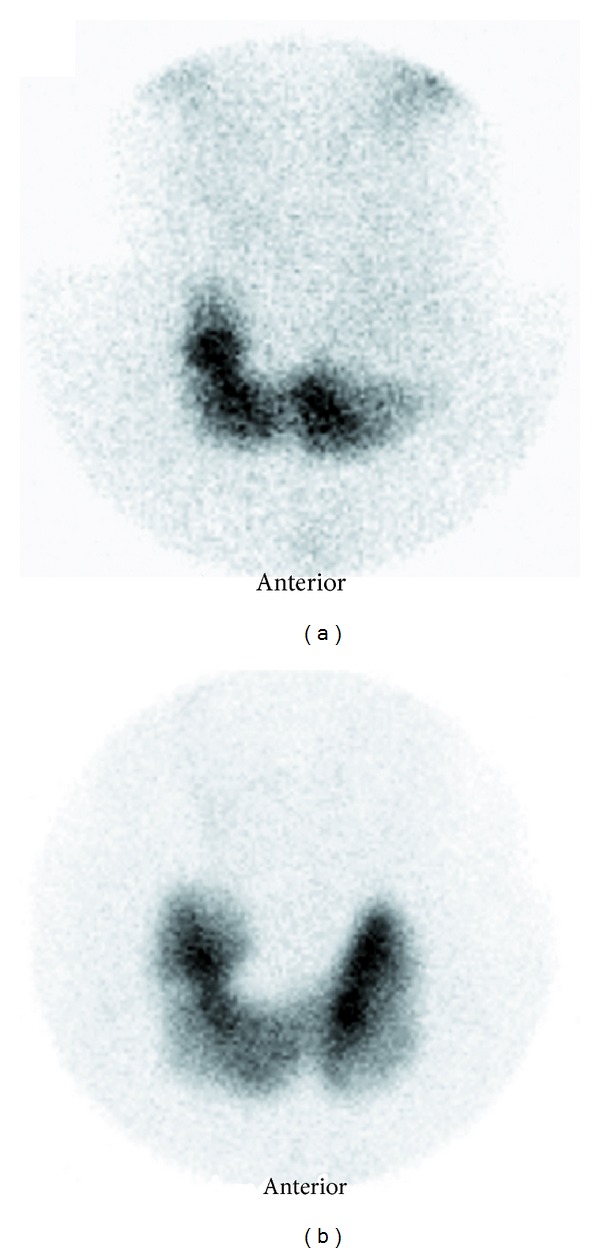
Thyroid scintigraphy using ^99m^Tc-pertechnetate. (a) Preoperative anterior planar image shows a large hypoactive area in upper and middle poles of the left thyroid lobe. (b) Postoperative anterior planar image demonstrates an increased focal uptake in the middle and upper poles of the left thyroid lobe.

Cervical lymph nodes had normal appearance. Tc-99m scintigraphy of thyroid gland was evaluated as a cold nodule occupying the middle and the superior regions of the left lobe (Figure 2(a)). 

**Figure 3:**
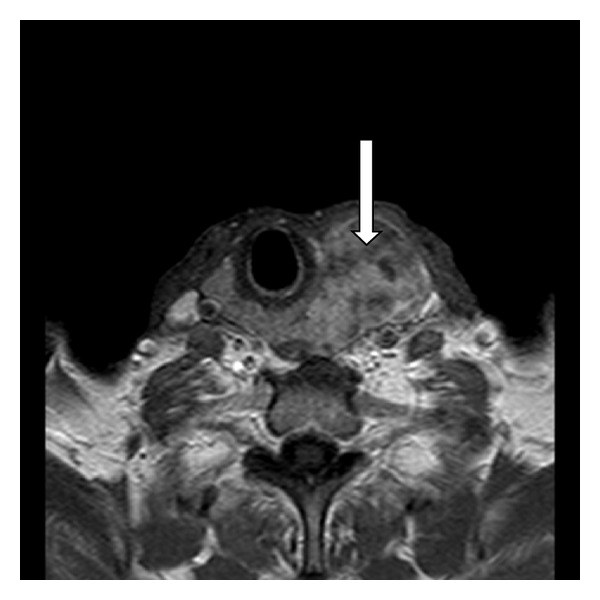
Magnetic resonance imaging scan showing heterogeneous mass in relation to the left thyroid lobe marked with an arrow.

An ultrasound-guided fine-needle aspiration biopsy (FNAB) of the lesion was performed. It was reported as suspicious probable malign cytology such as medullary thyroid carcinoma. The magnetic resonance imaging (MRI) showed a huge amorphous, heterogeneous, expansive mass occupying the space from the left lobe of the thyroid gland to the retrotracheal area, which was 6 × 8.5 × 8 cm in diameter with irregular margins from the thyroid gland (Figure 3). 

**Figure 4:**
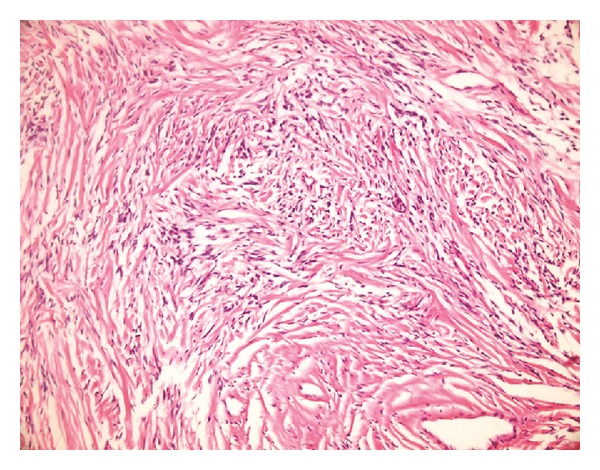
Patternless architecture and extensive stromal hyalinization (H&E ×200).

The patient underwent surgery for exploration and tumor excision. Macroscopically, the tumor was well circumscribed and externally compressing the left thyroid lobe. As the mass was evaluated as distinct lesion from the thyroid gland intraoperatively, left thyroid lobectomy was not performed. Histology showed the tumor composed of spindle cells with patternless architecture and extensive stromal hyalinization (Figure 4).

**Figure 5:**
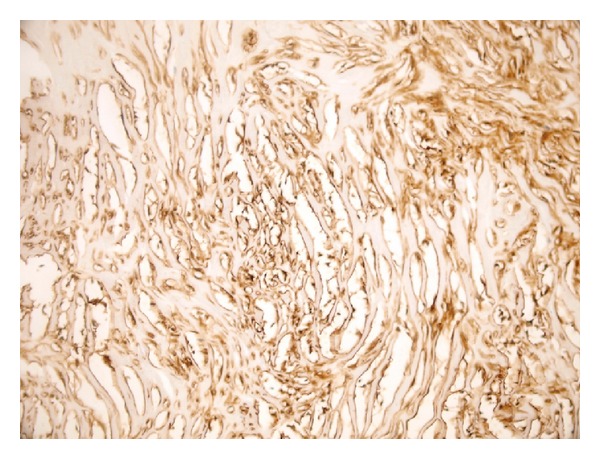
Diffuse CD34 staining in spindle cells (CD34 ×200).

**Figure 6:**
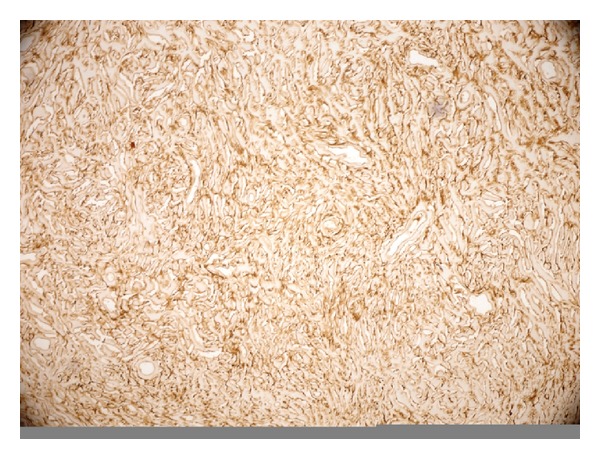
Diffuse CD34 staining in stroma (CD34 ×200).

**Figure 7:**
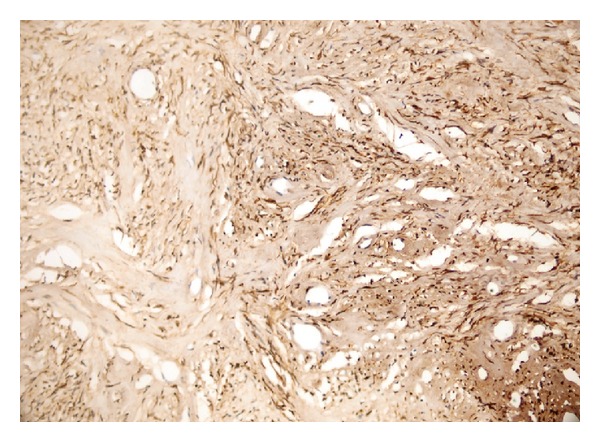
Diffuse bcl-2 staining (bcl-2 ×200).

The lesion had a strong positive immunohistochemical reaction for CD34 in spindle cells (Figure 5), stroma (Figure 6), and diffuse bcl-2 staining (Figure 7). 

The final pathological diagnosis was solitary fibrous tumor. There was no complication postoperatively. Nine months after resection, the patient was free of disease. Neck region was evaluated by ultrasound; the left lobe of thyroid was evaluated clearly. Postoperative thyroid scintigraphy showed increased focal uptake in the middle and superior poles of the left thyroid lobe (Figure 2(b)).

## 3. Discussion

SFT is a rare tumor in adults. It was first described as a pleura-based neoplasm or localized pleural mesothelioma [1, 12]. Since then, it has been reported that also extrapleural sites and almost any organ can be affected [13]. In 1991, Witkin and Rosai first described SFT of head and neck in a series of six cases [5]. Solitary fibrous tumor affecting the endocrine organs is uncommon. Among the endocrine organs, the thyroid gland [8, 9] is the most commonly involved organ, which is followed by pancreas [14], pituitary [15], and adrenal gland [16]. 

Symptoms of SFT in the neck are nonspecific and are related to general presence of a soft tissue mass in the area affected. They present nearly equally as asymptomatic, slowly enlarging masses or with local symptoms due to compression [6]. The period from the beginning of first symptoms to the diagnosis ranged from 2 months to 10 years in the literature for thyroid SFT [8]. For thyroid SFT, the patient age ranged from 28 to 70 years with a mean age of 50.5 years associated with slight female predominance [8]. The mean diameter of tumor was 48 ± 22 mm ranging from 15 mm to 97 mm [8]. 

There are some difficulties in differentiating perithyroidal soft tissue tumor from a mass belonging to thyroid. It may be confused with thyroid nodule [17] or goiter [18]. SFT of the thyroid gland typically represents itself as slowly enlarging cold nodules in glands [8]. However, as in our patient, rapidly growing nodules have been reported as isolated cases [7]. 

Thyroid SFT shows general benign clinical behavior. This data is in contrast to the outcome observed in patients with nonthyroidal SFT. Sung et al. reported malignancy rate as 30.2% for pleural SFTs [19]. Extrapleural SFTs mostly have a benign course, and malignant cases have been reported [20]. The present case should not be evaluated as original thyroid SFT. We recommend long-term followup of the patient as considering malignancy potential of nonpleural SFTs. 

Fine-needle aspiration biopsy is the gold standard method in differentiation of benign and malignant nodules in the thyroid gland [21]. FNAB experience is limited in thyroid SFT patients [8]. Parwani et al. described a patient with adequate material for a definite diagnosis of a spindle cell tumor before surgery [22]. In most patients, the procedure did not yield adequate material for a definite diagnosis [8]. Thyroid SFT should be differentiated from other thyroid lesions composed of spindle cells, including anaplastic carcinoma, spindle cell variant of medullary carcinoma, papillary carcinoma with nodular fasciitis-like stroma, benign and malignant mesenchymal tumors, and Riedel thyroiditis. Since FNAB was evaluated as suspicious for medullary thyroid carcinoma, in our case, we performed serum calcitonin level which was low, and also immunohistochemical calcitonin was negative before surgery.

All reported thyroid SFTs were histologically and immunohistochemically similar to benign SFTs in the pleura. Microscopically, they were composed of bland spindle fibroblast-like cells growing in patternless manner with variable amounts of intercellular collagen bundles, keloid-like hyalinization, and alternating hypercellular and hypocellular areas [23]. Immunohistochemically, benign SFTs generally showed strong positive reactions for CD34, vimentin, and bcl-2 but negative for SMA, desmin, and S-100 protein [11]. Among these markers, diffusely positive CD34 staining is the most important feature of SFTs [24]. In the present case, the tumor showed the same immunohistochemical profile. 

However, SFTs are generally considered to be benign neoplasm. Previous studies have demonstrated that 10–15% of intrathoracic SFTs were malignant [13]. In this study, histological features associated with malignancy were determined as follows: high cellularity, high mitotic activity (>4/10 HPF), atypical nuclear appearance, hemorrhage, and necrosis. In previous reports of thyroid SFT, almost all of the patients had a benign course, including one in which capsular invasion was observed [25]. Ning et al. reported the first case of malignant thyroid SFT in a 76-year-old woman with local recurrence and bilateral pulmonary metastasis [11]. Our patient's SFT did not have any criteria for malignancy. 

Computed tomography (CT) and MRI are often utilized in the assessment of SFTs to better define their extension into nearby tissues and preoperative evaluation. Because SFTs have presented themselves as huge masses, these imaging methods can demonstrate tracheal deviation [22, 24, 26] or adjacent tissue compression. The scintigraphic evaluation of the thyroid gland was performed in some of the previous thyroid SFT cases, and nodules were demonstrated as cold areas [7, 26]. In our case, tumor had contiguity and compression of the left lobe of the thyroid gland. It gave a cold nodule image in the thyroid scintigraphy and was confused with a thyroid nodule. After excision of the mass, this image was disappeared, and the left lobe was evaluated as normal in scintigraphy. 

## 4. Conclusion

Imaging techniques like magnetic resonance, ultrasonography, and thyroid scintigraphy may be insufficient for the neck masses on thyroid gland localization in order to distinguish its origin. For these patients, final diagnosis can be made by multidisciplinary approaches including the surgery. SFT associated with thyroid gland is very rare. Up to date, most patients had a benign course, but also malignant SFT of the thyroid was reported. However, our patient did not have any malignancy criteria. We recommend careful and long-term followup as clinical behavior of this rare tumor is still undetermined.
